# Effectiveness of Hypochlorous Acid to Reduce the Biofilms on Titanium Alloy Surfaces in Vitro

**DOI:** 10.3390/ijms17071161

**Published:** 2016-07-19

**Authors:** Chun-Ju Chen, Chun-Cheng Chen, Shinn-Jyh Ding

**Affiliations:** 1 Institute of Oral Science, Chung Shan Medical University, Taichung City 402, Taiwan; ccz1001@yahoo.com.tw; 2 School of Dentistry, Chung Shan Medical University, Taichung City 402, Taiwan; 3 Department of Dentistry, Chung Shan Medical University Hospital, Taichung City 402, Taiwan

**Keywords:** dental implant, hypochlorite acid, antiseptics, antimicrobial activity

## Abstract

Chemotherapeutic agents have been used as an adjunct to mechanical debridement for peri-implantitis treatment. The present in vitro study evaluated and compared the effectiveness of hypochlorous acid (HOCl), sodium hypochlorite (NaOCl), and chlorhexidine (CHX) at eliminating Gram-negative (*E. coli* and *P. gingivalis*) and Gram-positive (*E. faecalis* and *S. sanguinis*) bacteria. The effect of irrigating volume and exposure time on the antimicrobial efficacy of HOCl was evaluated, and a durability analysis was completed. Live/dead staining, morphology observation, alamarBlue assay, and lipopolysaccharide (LPS) detection were examined on grit-blasted and biofilm-contaminated titanium alloy discs after treatment with the three chemotherapeutic agents. The results indicated that HOCl exhibited better antibacterial efficacy with increasing irrigating volumes. HOCl achieved greater antibacterial efficacy as treatment time was increased. A decrease in antimicrobial effectiveness was observed when HOCl was unsealed and left in contact with the air. All the irrigants showed antibacterial activity and killed the majority of bacteria on the titanium alloy surfaces of biofilm-contaminated implants. Moreover, HOCl significantly lowered the LPS concentration of *P. gingivalis* when compared with NaOCl and CHX. Thus, a HOCl antiseptic may be effective for cleaning biofilm-contaminated implant surfaces.

## 1. Introduction

The use of dental implants to replace missing teeth has provided predictable and good long-term results [1,2,3,4]. However, the incidence of short- and long-term complications has increased. The two most common reasons for implant failure are overloading and infection. It is generally accepted that microbial biofilms play a significant role in the development of peri-implant diseases [5,6]. When the pristine surface of a dental implant is exposed to the oral cavity following installation, bacterial colonization takes places in a manner similar to that of teeth, which in turn gives rise to the development of mature biofilms [7,8]. Removal of the bacteria and their byproducts, such as LPS, has proven problematic [9] because of the screw-shaped design and surface microstructure of implants. Mechanical debridement alone is incapable of removing all biofilms. In addition, implants with rough surfaces and a large surface free energy tend to accumulate more plaque [10]. Infections are difficult to eliminate because the initial bacterial adhesion starts in areas of high wettability and inside the pits and grooves of the roughened surfaces [11]. Thus, the use of different chemotherapeutic agents has been proposed for the treatment of infected implant surfaces. To decrease the number of microorganisms to a level compatible with periodontal health, clinicians often use antimicrobial agents, such as CHX, NaOCl, citric acid, hydrogen peroxide, and tetracycline paste, as an adjunctive treatment to mechanical debridement [12,13,14]. However, satisfying therapeutic strategies or scientifically based treatment recommendations remain unavailable [14].

The use of high doses of an antimicrobial agent is effective at eradicating bacteria. Zablotsky et al. found that among the antiseptics tested for treating contaminated titanium surfaces, citric acid resulted in the lowest amounts of residual LPS when compared with the saline control; however, these differences failed to reach statistical significance [9]. Dennison et al. studied decontamination treatments for LPS (from *P. gingivalis*)-contaminated machined and titanium plasma-sprayed (TPS) implants using deionized water, citric acid solution, and 0.12% CHX [15]. The citric acid and CHX treatment presented no statistically significant differences in antibacterial effectiveness on machined and TPS surfaces when compared with deionized water. Commonly used antiseptics are effective at killing microorganisms but not removing biofilm on titanium implants [13]. Biofilms are heterogeneous structures that have a high resistance to antimicrobial agents [16].

In addition to the aforementioned antiseptics, HOCl has attracted attention. HOCl is generated by the body’s immune cells to fight invading pathogens and infection [17]. At an effective antimicrobial concentration range, HOCl is non-irritating and non-sensitizing because of its lower cytotoxicity to mammalian cells when compared with NaOCl and H2O2 [18]. In dental treatment procedures, a mild acidic HOCl solution is introduced as an endodontic irrigating solution [19] and as a chemotherapeutic agent for the treatment of periodontitis [20]. However, a search of the literature shows a paucity of articles addressing the effects of HOCl as a treatment for bacterial growth on titanium implants. We hypothesized that HOCl would be effective at eradicating bacteria on implant surfaces in vitro. Accordingly, the purpose of the present study was (1) to examine the effectiveness of HOCl at different irrigating volumes and treatment times at eliminating Gram-positive and Gram-negative bacterial strains; and (2) compare the effects of HOCl, NaOCl and CHX on biofilm-contaminated titanium alloy surfaces used as a model of peri-implantitis. Importantly, LPS concentrations remaining on the titanium alloy surfaces were examined because the biofilms inactivated/killed by irrigants may remain on the implant surface, which would facilitate subsequent microbial adhesion.

## 2. Results

### 2.1. Antibacterial Effectiveness of HOCl

#### 2.1.1. HOCl Volume Effect

**Figure 1:**
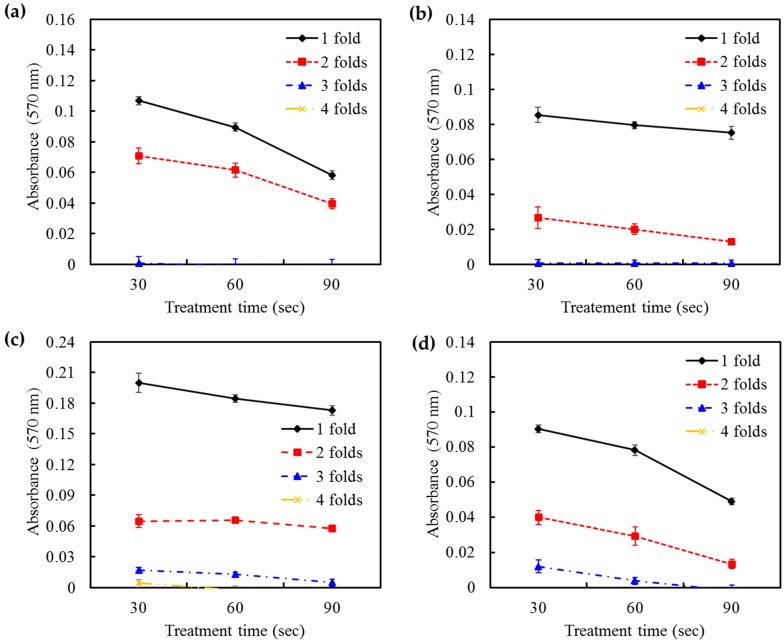
The antimicrobial effect of hypochlorous acid (HOCl) at different volume ratios and treatment times on (**a**) *E. coli*; (**b**) *P. gingivalis*; (**c**) *E. faecalis*; and (**d**) *S. sanguinis*. The results were reported in absorbance units. HOCl showed volume-dependent antibacterial ability against all four species. The antibacterial efficacy of HOCl was also time-dependent.

Clinical significance is the practical importance of treatment efficacy. The antibacterial activity of HOCl at different volume ratios to bacterial suspensions of *E. coli*, *P. gingivalis*, *E. faecalis*, and *S. sanguinis* is shown in Figure 1. As expected, the antibacterial ability of HOCl against the four strains was volume-dependent. Notably, the 4:1 volume ratio of HOCl to bacterial solution completely killed the bacteria. This result was similar to the effectiveness of 1.3% NaOCl and 0.2% CHX at a 1:1 volume ratio with a 30 s treatment time. The results of 1.3% NaOCl and 0.2% CHX were not shown because of undetectable absorbance values. As the treatment time increased, HOCl showed significantly greater antibacterial efficacy (*p* < 0.05). Based on these data, subsequent experiments used a 4:1 volume ratio of the irrigant (HOCl, NaOCl, and CHX) to bacterial suspension.

#### 2.1.2. HOCl Durability

**Figure 2:**
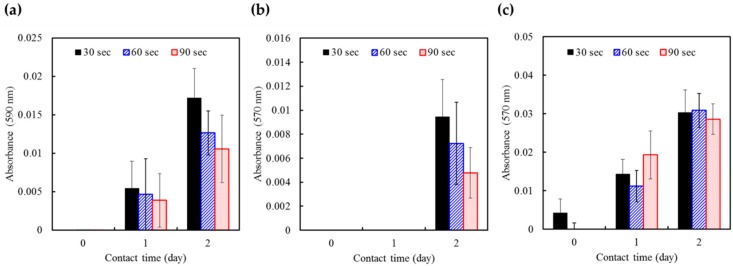
The changes in the antimicrobial effectiveness of HOCl as a function of contact time with air on (**a**) *E. coli*; (**b**) *P. gingivalis*; and (**c**) *E. faecalis*. The volume ratio of HOCl to bacterial suspension was 4:1. The results were reported in absorbance units. The duration of air contact adversely affected the antimicrobial activity of HOCl; however, the treatment duration did not have a significant effect.

The durability of irrigants is an essential parameter in the evaluation of antimicrobial efficacy. To determine the durability of HOCl, the irrigant was unsealed and left in contact with the air at ambient temperature for 1 or 2 days. Next, the antibacterial activity against the four bacteria was detected. The changes in antimicrobial effectiveness of HOCl as a function of contact time are presented in Figure 2. The results of *S. sanguinis* were not shown because of undetectable values. HOCl showed a time-dependent loss of antimicrobial effectiveness when left in direct contact with the air. Interestingly, the statistical analysis showed that a treatment time in the range of 30–90 s did not influence the antimicrobial effectiveness of HOCl against bacteria. On the other hand, the pH values of the HOCl solution after contact with the air for 1 day and 2 days were 6.85 and 6.91, respectively. Both of these values were significantly higher (*p* < 0.05) than the original pH value of 6.73. Notably, the pH approached stability and held at 6.95 for an extended contact time of up to 7 days.

### 2.2. Antibacterial Activities on Titanium Alloy

#### 2.2.1. Live/Dead Staining

**Figure 3:**
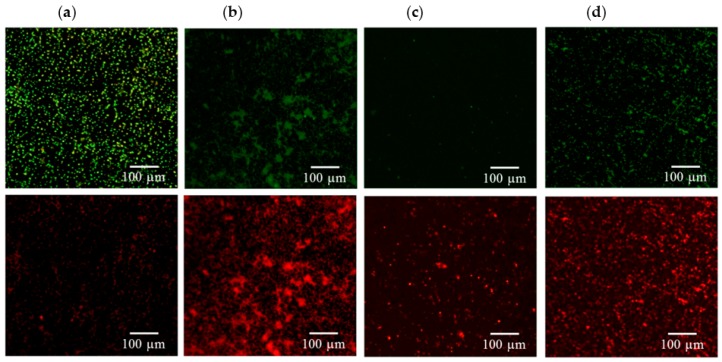
Viability staining of (**a**) *E. coli* exposed to (**b**) HOCl, (**c**) sodium hypochlorite (NaOCl), and (**d**) chlorhexidine (CHX). Viable bacteria are labeled green, and dead bacteria are labeled red. Fewer viable *E. coli* bacteria were found after treatment with the three irrigants.

**Figure 4:**
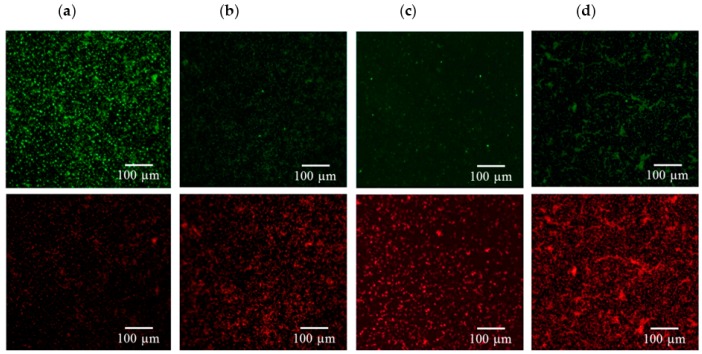
Viability staining of (**a**) *P. gingivalis* exposed to (**b**) HOCl, (**c**) NaOCl, and (**d**) CHX. Viable bacteria are labeled green, and dead bacteria are labeled red. The three irrigants induced significant reductions in bacterial numbers when compared with the control.

**Figure 5:**
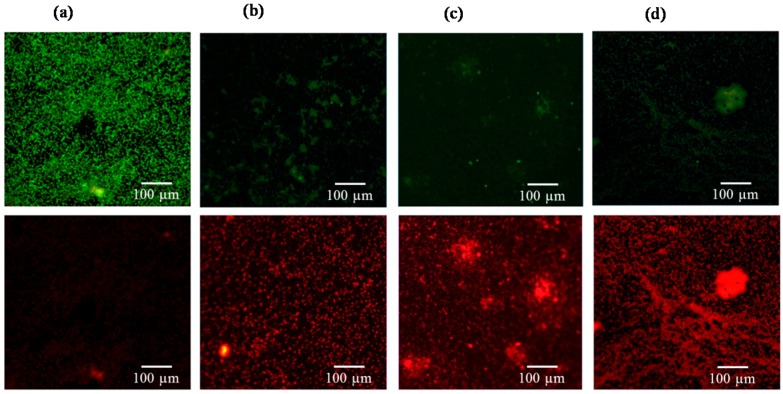
Viability staining of (**a**) *E. faecalis* exposed to (**b**) HOCl, (**c**) NaOCl, and (**d**) CHX. Viable bacteria are labeled green, and dead bacteria are labeled red. The dead bacteria in the irrigant-treated groups made up a greater proportion of the total bacteria on the titanium alloy surfaces when compared with the control.

**Figure 6:**
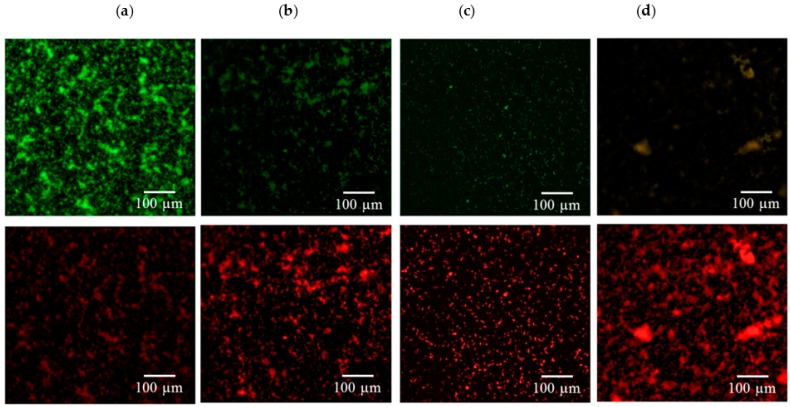
Viability staining of (**a**) *S. sanguinis* exposed to (**b**) HOCl, (**c**) NaOCl, and (**d**) CHX. Viable bacteria are labeled green, and dead bacteria are labeled red. An increased amount of dead bacteria was observed after irrigant treatment.

Figure 3, Figure 4, Figure 5 and Figure 6 consistently indicate that all the irrigants cause the majority of bacteria on the titanium alloy surfaces to turn red in the live/dead cell assay when compared with the unirrigated control. This result demonstrated that HOCl exerted an antibacterial activity that was similar to NaOCl and CHX.

#### 2.2.2. SEM Observation

**Figure 7:**
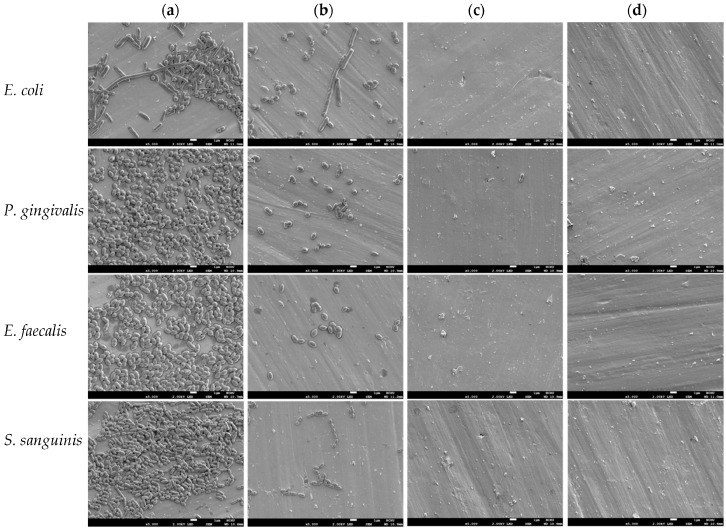
Scanning electron micrographs of (**a**) the four bacterial species exposed to (**b**) HOCl, (**c**) NaOCl, and (**d**) CHX showed that the three irrigants appreciably reduced the number of bacteria. Almost a complete removal of the bacteria adhered to the surface was observed after treatment with NaOCl and CHX. Scale bar, 1 μm.

To clarify the effect of irrigants on the number of bacteria on the titanium alloy surfaces, the surface morphologies of the samples were observed using SEM before and after irrigant treatment. Although the rod-shaped *E. coli* was contaminated during incubation with the appearance of differently shaped bacteria, the number of *E. coli* bacteria was appreciably reduced after irrigant treatment (Figure 7). Similarly, the three irrigants effectively reduced the number of the other three bacterial species when compared with the unirrigated control. In contrast to the findings in the HOCl-treated groups, almost a complete removal of bacterial species adhered to the surface was observed after treatment with NaOCl and CHX.

#### 2.2.3. AlamarBlue Assay

**Figure 8:**
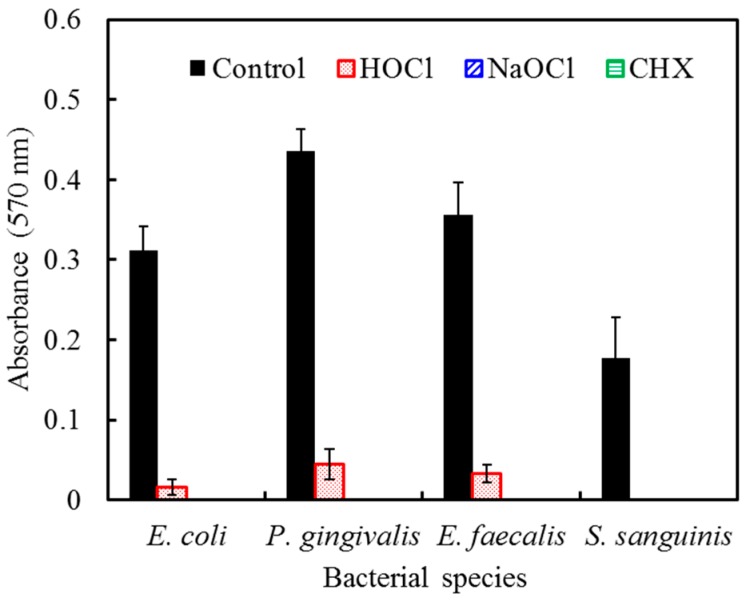
Antimicrobial effectiveness of HOCl, NaOCl, and CHX against *E. coli*, *P. gingivalis*, *E. faecalis*, and *S. sanguinis* on titanium alloy surfaces after culture for 24 h. The data were presented as absorbance units. Notably, all the irrigants significantly eliminated bacterial adhesion.

To further clarify bacterial viability on the implant surfaces, an alamarBlue assay was used to examine the antimicrobial effectiveness of HOCl, NaOCl, and CHX. The three irrigants effectively eliminate bacteria (Figure 8). The antimicrobial activity of HOCl was slightly inferior to NaOCl and CHX, which is consistent with our SEM observations (Figure 7).

#### 2.2.4. LPS Detection

**Figure 9:**
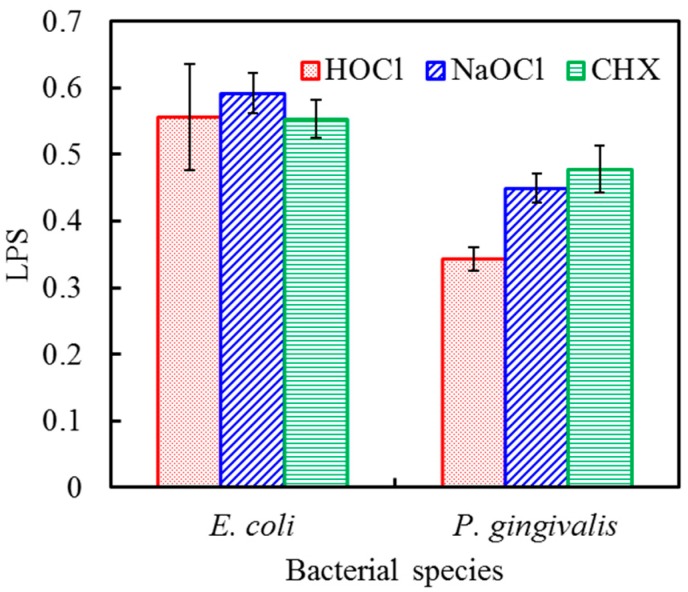
Residual lipopolysaccharide (LPS) levels from *E. coli* and *P. gingivalis* on titanium alloy surfaces after HOCl, NaOCl, and CHX treatment for 60 s. There was no significant difference in the residual LPS levels from E. coli. However, treatment with HOCl caused a significant decrease in the LPS from *P. gingivalis* when compared with NaOCl and CHX.

LPS, also termed endotoxin, is the major cell wall component of Gram-negative bacteria, such as *E. coli* and *P. gingivalis*. To investigate the removal of biofilms from the titanium alloy surfaces, the amount of residual LPS on the implant surface was examined after treatment with the different antiseptics. Figure 9 shows no significant difference (*p* > 0.05) in residual LPS levels from *E. coli* after HOCl, NaOCl, and CHX treatment for 60 s. In contrast to these findings, HOCl produced a significant increase (*p* < 0.05) in the removal of LPS from *P. gingivalis* when compared with NaOCl and CHX.

## 3. Discussion

Implant-associated infections are due to bacterial biofilms that form on the implant surface; thus, the bacterial colonization of implants can never be completely avoided [21]. The possible reasons for biofilm formation include intraoperative contamination, systemic spreading and permanent transcutaneous passages [22]. In this study, four different bacterial species were used to examine the efficacy of chemotherapeutic agents. *E. coli* is commonly used as a Gram-negative model organism. *P. gingivalis* is associated with periodontitis and also implicated in peri-implantitis [23]. Of the endodontic pathogens, *E. faecalis* has been extensively studied because this species is frequently found in endodontic infections [24]. *S. sanguinis* is often present in the human oral cavity and known as a pioneer bacterium of oral biofilms [14]. The most frequently used chemotherapeutic agents for the treatment of endodontic and periodontal infections are CHX and NaOCl [25]. Chemical therapy creates only a minimal risk of damage to the titanium implant surface. Nevertheless, in vivo studies have failed to identify one chemotherapeutic agent as the gold standard for implant surface decontamination [26]. The frequently used concentration of commercially available CHX is 0.12%; however, this dose did not achieve a significant reduction in LPS on contaminated titanium surfaces when compared with untreated controls [27]. In a study by Dennison et al. [15], a 0.12% CHX treatment removed 94.6% of the LPS from contaminated machined implant surfaces, but only 37.1% of the LPS was removed from contaminated plasma-sprayed implant surfaces. Thus, CHX was only modestly effective at removing biofilms. In the present study, the high antimicrobial effectiveness and low LPS removal ability of CHX on titanium alloy confirmed the findings from previous studies [15,27].

NaOCl has a broad spectrum of antimicrobial activity [25]. However, NaOCl may be harmful because it is extremely alkaline, irritating and cytotoxic at the concentrations typically used [25,27]. The antimicrobial activity of NaOCl is dependent on the concentration of un-dissociated HOCl in solution, and the hypochlorite ion (OCl^−^) is less effective than un-dissociated HOCl [25]. Indeed, commercial NaOCl solutions exhibit less antimicrobial potential than mildly acidic HOCl solutions within its effective antimicrobial concentration range [28]. Our results showed that HOCl failed to be equivalent to the efficacy of NaOCl and CHX at the same volume and exposure time. In fact, the used concentration of HOCl (0.018%) was much lower than those of NaOCl (1.3%) and CHX (0.2%) even if 4-fold volume was used. A 4:1 volume ratio of 180 ppm (0.018%) HOCl to bacterial solution completely killed all four of the bacterial strains. This level of antimicrobial efficacy was equivalent to the 1:1 volume ratio of 1.3% NaOCl and 0.2% CHX, although their absorbance below the detectable range. Our finding on the volume dependence of the antimicrobial activity of HOCl is consistent with a previous study [25]. According to the literature [24], the treatment time required to eliminate bacteria appreciably depends on the concentration (or volume) and type of irrigant used. A significantly higher concentration of the antiseptic and longer treatment time is required to completely kill bacteria [29].

Pure HOCl has not been developed as a commercial pharmaceutical formulation presumably because of the challenge of maintaining storage stability [18]. We demonstrated that the antimicrobial efficacy of HOCl significantly decreased when HOCl was left in an open environment in contact with the air at ambient temperature. This is a potential problem for the clinical use of HOCl. The dissociation of HOCl to the less microbicidal OCl^−^ depends on pH. At an approximate pH of 6, the concentration of HOCl is optimal and its dissociation is minimal [18,30]. As the pH increases, more OCl^−^ is formed [31]. HOCl was reported to have an antimicrobial effect approximately 80–100 times stronger than the OCl^−^ ion [19], which can explain our results that showed a decrease in the antimicrobial activity of HOCl after contact with the air. Stabilized HOCl displayed rapid and concentration-dependent activity against clinically relevant microorganisms as long as the effective pH range was maintained [29]. To stabilize the balanced HOCl solution, it should be stored in a tightly sealed container. Thus, HOCl could be a durable antibacterial irrigant in daily clinical use if handled properly.

A critical pathogenic event in the process of biofilm formation is bacterial adhesion on the titanium surface [32]. The effect of residual dead bacteria on the healing process of previously contaminated implants and the extent of bacterial removal required to achieve a successful treatment outcome requires further investigation [1,33]. In the present study, we used a simple grit-blasted titanium alloy to create biofilms for subsequent tests with antimicrobial agents. Based on the preliminary results, we further compared the antibacterial efficacy of HOCl, NaOCl, and CHX on titanium alloy surfaces using a 4:1 volume ratio and 60 s exposure time, and focused on the detection of residual LPS. In the case of *E. coli*, the three antiseptics revealed no significant differences in the levels of residual LPS on the titanium alloy surface. The 1.3% NaOCl and 0.2% CHX solutions demonstrated slightly higher antimicrobial activity than 0.018% HOCl in the alamarBlue assay. However, the 0.018% HOCl resulted in a greater reduction of LPS from *P. gingivalis*-contaminated titanium alloy surfaces when compared with 1.3% NaOCl and 0.2% CHX. LPS are large molecules that consist of a lipid and an O-antigen-based polysaccharide. HOCl forms chlorohydrins by attacking the double bonds of unsaturated lipids [34], and chlorohydrins have the potential to damage cell membranes and induce cytolysis [35]. The results of this in vitro study demonstrated that it is possible to significantly reduce biofilm-associated bacterial populations in a clinically relevant time period. However, the mechanism of LPS removal by HOCl requires further investigation.

## 4. Materials and Methods

### 4.1. Irrigants and Microorganisms

The tested irrigant was 180 ppm (0.018%) HOCl liquid (Superclean) kindly donated from Union Biomedical Corporation (New Taipei City, Taiwan). Two commonly used irrigants, 1.3% NaOCl (Shimakyu’s Pure Chemical, Osaka, Japan) and 0.2% chlorhexidine gluconate (Panion & BF Biotech, Taoyuan, Taiwan), were used for comparative purposes. In this study, Gram-negative bacteria strains (*Escherichia coli* (*E. coli*, ATCC DH5 ALPHA) and *Porphyromonas gingivalis* (*P. gingivalis*, A7436)) and Gram-positive bacteria strains (*Enterococcus faecalis* (*E. faecalis*, ATCC 29212) and *Streptococcus sanguinis* (*S. sanguinis*, ATCC 10556)) were used. The bacteria were cultivated in Wilkins-Chalgren Anaerobe broth (Oxoid, Hampshire, UK) broth at 37 °C under anaerobic conditions.

### 4.2. Antibacterial Effectiveness of HOCl

The antibacterial effectiveness of various volumes of HOCl on bacterial strains was determined using an alamarBlue (Invitrogen, Grand Island, NY, USA) assay that was used for real-time and repeated monitoring of bacterial viability. Volumes of 1, 2, 3, or 4 mL of 180 ppm HOCl was added to 1 mL of a bacterial suspension solution containing approximately 105 colony-forming units (CFU) for 30, 60 or 90 s. One mL of 1.3% NaOCl and 0.2% CHX were also used according to this method. After the end of the treatment, alamarBlue was added to the test solution and incubated at 37 °C for 20 min. The solution in each tube was transferred to a new 96-well tissue culture plate. Plates were read in a Sunrise Microtiter reader (Tecan Austria Gesellschaft, Salzburg, Austria) at 570 nm with a reference wavelength of 600 nm. The results were reported in absorbance units. The number of living bacteria was estimated from the redox reactions between the indicator dye and metabolically active bacteria. The maximum absorption value at 570 nm is directly proportional to the number of live bacteria. The absorbance results were obtained in nine independent measurements. In addition, HOCl was unsealed and left in contact with the air for 1 or 2 days at ambient temperature. To examine its durability, 4 mL of the unsealed HOCl was then used in the alamarBlue assay to examine changes in antibacterial efficacy. The data provided for each group are the means of nine independent measurements. The pH values of the unsealed HOCl solution were measured using a pH meter (Suntex SP-701, Taipei, Taiwan). The analysis was carried out in three separate sets of experiments.

### 4.3. Preparation of Titanium Alloy

Commercially available 3 mm-thick Ti-6Al-4V alloys (ASTM F136-84; Titanium Industries, Parsippany, NJ, USA) of 10 × 10 mm^2^ were selected as substrate material. All samples were wet-ground with 1200-grit SiC abrasive paper (3M Wetordry TriMite 734, St. Paul, MN, USA), ultrasonically cleaned in acetone and ethanol for 20 min, rinsed in deionized water, and then dried in an oven at 60 °C.

### 4.4. Live/Dead Staining on Titanium Alloy

One milliliter of each bacterial suspension at a density of 10^5^ CFU/mL was placed on the titanium alloy surface in 24-well culture plates. The samples were incubated at 37 °C for 24 h for subsequent analyses, including live/dead staining, morphology observation, alamarBlue assay, and LPS detection. The choice of 24 h was based on the time interval for the stationary phase of microbial growth curves. For the live/dead fluorescent stain, the samples were washed twice with phosphate buffer solution (PBS, pH 7.4). Next, HOCl, NaOCl, and CHX were added to the plates at a volume 4 times that of the bacterial suspension. Samples were allowed to react at room temperature for 60 s. The samples were then washed with deionized water and incubated with the BacLight Live/Dead bacterial viability solution (Molecular Probes, Eugene, OR, USA) for 15 min. The staining reagent was removed with deionized water, and the bacteria adhered on the specimens were visualized using a ZEISS AXioskop2 microscope (Carl Zeiss, Thornwood, NY, USA) at 200× magnification.

### 4.5. Morphology Observation on Titanium Alloy

After exposure to the irrigants, the samples were washed three times with PBS and fixed in 2% glutaraldehyde (Sigma, St. Louis, MO, USA). The samples were then dehydrated using a graded ethanol series for 20 min at each concentration. The dried samples were mounted on stubs, coated with a gold layer, and viewed using scanning electron microscopy (SEM; JEOL JSM-7800F, Tokyo, Japan).

### 4.6. AlamarBlue Assay on Titanium Alloy

After treatment of the bacteria with irrigants, the solution was discarded and the plate was filled with alamarBlue reagent. The assay was performed as previously stated in the Methods section. The data shown are the means from fifteen parallel experiments.

### 4.7. LPS Detection on Titanium Alloy

After the irrigants reacted with the bacterial solutions and samples were washed with PBS twice, the remaining LPS concentrations on the substrates were quantified using a ToxinSensor chromogenic Limulus Amebocyte Lysate endotoxin assay kit (GenScript, Piscataway, NJ, USA) according to the manufacturer’s instructions. Five replicates were carried out for each group, and the results are expressed as the mean ± standard deviation.

### 4.8. Statistical Analysis

All the results are expressed as the mean ± standard derivation for the total number of replicate experiments indicated unless otherwise stated. A one-way analysis of variance (ANOVA) was used to evaluate significant differences between the means. Scheffe’s multiple comparisons were used to determine the significance of the standard deviations between the sample measurements under different experimental conditions. The result was considered statistically significant when the *p*-value was less than 0.05.

## 5. Conclusions

Within the limitations of this in vitro model, HOCl is effective for cleaning biofilm-contaminated implant surfaces and has the potential to be an antiseptic for peri-implantitis treatment. These results suggest that the efficacy of HOCl is equivalent to NaOCl and CHX. The concentration of HOCl (0.018%) was lower than those for NaOCl (1.3%) and CHX (0.2%) but still showed efficacy against the four bacterial species. Notably, HOCl was superior to the other antiseptics in the reduction of residual LPS from *P. gingivalis* on implant surfaces. However, additional studies, including use of the multispecies biofilm model and screw-shaped implants and other biocompatibility tests, are required prior to the clinical application of HOCl as an antiseptic.
